# Recent progress in MnBi_2*n*_Te_3*n*+1_ intrinsic magnetic topological insulators: crystal growth, magnetism and chemical disorder

**DOI:** 10.1093/nsr/nwad282

**Published:** 2023-11-14

**Authors:** Chaowei Hu, Tiema Qian, Ni Ni

**Affiliations:** Department of Physics and Astronomy and California NanoSystems Institute, University of California-Los Angeles, Los Angeles, CA 90095, USA; Department of Physics and Astronomy and California NanoSystems Institute, University of California-Los Angeles, Los Angeles, CA 90095, USA; Department of Physics and Astronomy and California NanoSystems Institute, University of California-Los Angeles, Los Angeles, CA 90095, USA

**Keywords:** MnBi_2_Te_4_, magnetic topological insulator, magnetism tuning, antiferromagnetism, ferromagnetism, pressure, chemical doping

## Abstract

The search for magnetic topological materials has been at the forefront of condensed matter research for their potential to host exotic states such as axion insulators, magnetic Weyl semimetals, Chern insulators, etc. To date, the MnBi_2*n*_Te_3*n*+1_ family is the only group of materials showcasing van der Waals-layered structures, intrinsic magnetism and non-trivial band topology without trivial bands at the Fermi level. The interplay between magnetism and band topology in this family has led to the proposal of various topological phenomena, including the quantum anomalous Hall effect, quantum spin Hall effect and quantum magnetoelectric effect. Among these, the quantum anomalous Hall effect has been experimentally observed at record-high temperatures, highlighting the unprecedented potential of this family of materials in fundamental science and technological innovation. In this paper, we provide a comprehensive review of the research progress in this intrinsic magnetic topological insulator family, with a focus on single-crystal growth, characterization of chemical disorder, manipulation of magnetism through chemical substitution and external pressure, and important questions that remain to be conclusively answered.

## Teaser

This review focuses on the crystal growth, disorders, and tunable magnetism through chemical doping and pressure in MnBi_2*n*_Te_3*n*+1_, providing valuable insights into the design, development, and defect control of MnBi_2*n*_Te_3*n*+1_ and other potential intrinsic magnetic topological insulators.

## INTRODUCTION

The discovery of the magnetic topological insulator MnBi_2_Te_4_ has been a major advancement in the field of condensed matter physics in its pursuit of the high-temperature quantum anomalous Hall effect (QAHE) [1–5]. A robust quantized Hall resistivity is found at the record high temperature of 1.4 K under a zero field, and even higher temperatures under an external field [4–6], making QAHE much more accessible for its promising applications in low-energy-consumption devices, quantum metrology and quantum computing [7–9]. The report of QAHE in MnBi_2_Te_4_ follows a decade of precedent works after the discovery of topological insulators. The interplay of non-trivial band topology and magnetic order can lead to numerous novel topological states, such as axion insulators, magnetic Weyl semimetals, Chern insulators and three-dimensional quantum anomalous Hall (QAH) insulators [10]. This has motivated an intensive ongoing search for materials capable of hosting these proposed states. In 2013, QAHE was first achieved below 90 mK in molecular-beam epitaxy-grown thin films of Cr_0.15_(Bi_0.1_Sb_0.9_)_1.85_Te_3_ [11]. The unavoidable magnetic inhomogeneity introduced by magnetic dopants is believed to account for the low critical temperature of the QAHE. Therefore, a magnetic topological insulator (MTI) with intrinsic magnetism is strongly urged. In 2018, MnBi_2_Te_4_ was discovered as the first van der Waals (vdW) material that features intrinsic magnetism and non-trivial band topology [1–5,12–25]. It has an A-type antiferromagnetic (AFM) structure where the spins are aligned ferromagnetically in plane and antiferromagnetically out of plane. Its vdW nature makes possible the exfoliation of the bulk crystal into a thin-film device. As such, one can get even-layer two-dimensional (2D) AFM devices and odd-layer 2D devices with net ferromagnetic (FM) moments. In addition, both angle-resolved photoemission spectroscopy (ARPES) and first-principles density functional theory (DFT) reveal a clean band structure where only the topological surface state appears at the charge neutrality point (CNP) [1,2,20]. Altogether, these unique properties facilitated the rapid realization of QAHE and various other emergent topological states in the MnBi_2_Te_4_ devices [4–6,26,27].

**Figure 1.**
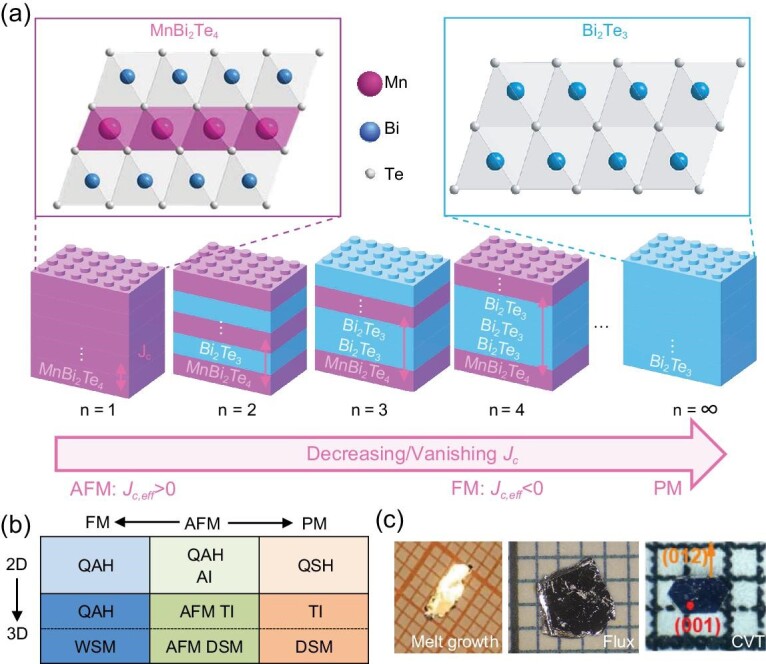
(a) A ‘lego set’ of MnBi_2*n*_Te_3*n*+1_ and the side view of the [MnBi_2_Te_4_] SL and [Bi_2_Te_3_] QL. Purple block: [MnBi_2_Te_4_] SL. Blue block: [Bi_2_Te_3_] QL. (b) Rich topological quantum states proposed in *M*Bi_2_Te_4_ (*M* = transition/rare-earth metal) in two and three dimensions under different magnetic states from [2]. AI, axion insulator; WSM, Weyl semimetal; DSM, Dirac semimetal. (c) Pictures of single crystals grown from melt growth [57], Bi_2_Te_3_ flux [15] and CVT [58], respectively.

Following the proposal of MnBi_2_Te_4_ as an intrinsic MTI, other natural heterostructural members of the MnBi_2*n*_Te_3*n*+1_ (*n* ≥ 2) series were discovered [13,25,28–47]. The extension of MnBi_2_Te_4_ to the greater natural heterostructural MnBi_2*n*_Te_3*n*+1_ family can be seen as a ‘lego set’ with alternating ‘lego pieces’ of one MnBi_2_Te_4_ septuple layer (SL) and (*n* − 1) Bi_2_Te_3_ quintuple layers (QLs) along the *c* axis, as shown in Fig. 1(a). They are all reported to be intrinsic MTIs with clean band structures near the CNP.

With the natural heterostructure design, various magnetic topological states are theoretically proposed in MnBi_2*n*_Te_3*n*+1_. Figure 1(b) summarizes the proposed topological states in *M*Bi_2_Te_4_ (*M* = transition/rare-earth metal) [2] depending on the relative strength of spin-orbit coupling and magnetic interactions. Among these topological phases, experimental evidence of Chern insulator and AFM axion insulator states in 2D devices [4–6], as well as AFM TI, PM TI and FM Weyl semimetal states in 3D bulk MnBi_2_Te_4_ have been observed [48–50]. When the family is extended to MnBi_2*n*_Te_3*n*+1_, the overall magnetic interaction is weaker, so the magnetic state is more tunable and the topology can be modified accordingly. The AFM members of high-*n* MnBi_2*n*_Te_3*n*+1_ are AFM TIs [28,29,35], whereas the FM members of high-*n* MnBi_2*n*_Te_3*n*+1_ are FM axion insulators where the top and bottom surfaces each contributes half-quantized Hall conductance [30,51]. In addition, the vdW nature of high-*n* MnBi_2*n*_Te_3*n*+1_ also allows the fabrication of new heterostructures that were proposed to give rise to novel topological states, for example, the quantum spin Hall effect with broken time-reversal symmetry in the QL/SL/QL structure [28]. Therefore, this unique natural heterostructural intrinsic MTI series has inspired intensive investigations of their magnetism, band topology, chemical disorder, device functionality, etc.

There are mainly two general ways to obtain MnBi_2*n*_Te_3*n*+1_ flakes: the bottom-up approach that uses molecular-beam epitaxy (MBE) to grow the thin film layer by layer, and the top-down approach that grows single-crystal form and mechanically exfoliates the crystal down to the atomic limit. The MBE allows one to access more metastable phases such as MnBi_2*n*_Se_3*n*+1_ and Mn_4_Bi_2_Te_7_ [52–54] and control local defects, although it is difficult to get rid of the Mn-doped Bi_2_Te_3_ impurities [55]. The *in situ* measurements such as *in situ* scanning tunneling microscopy (STM) and ARPES allow the study of the band structure, topology and defects without sample degradation that may occur during the device fabrication process [56].

On the other hand, many experimental techniques still require the material in its single-crystal form because a single crystal offers a large volume, well-defined edges and surfaces, crystal orientations, reduced defect levels and uniformity over a large area. The QAHE report was reported in an exfoliated five-layer MnBi_2_Te_4_ device [4]. Therefore, high-quality single crystals with minimized defects, reduced magnetic inhomogeneity, low carrier density and high mobility are crucial for the studies of MnBi_2*n*_Te_3*n*+1_ and the realization of various emergent phenomena.

Despite rapid advancements in theoretical comprehension and experimental methodologies, research pertaining to MnBi_2*n*_Te_3*n*+1_ still faces big challenges. For example, even though quantized Hall conductance has been observed above 8 T in a few groups worldwide, achieving QAHE at zero field has only been realized in one group. Therefore, there exists a pressing need for endeavors to improve crystal growth techniques, enhance device fabrication processes and deepen our understanding of how defects affect magnetism and band topology. These collective efforts are imperative to enhance the success rate of attaining the zero-field QAH state. This review, as an integral component of the Special Topic titled ‘Recent Progress on MnBiTe Intrinsic Topological Magnetic Materials’ will thus be dedicated to crystal growth, chemical disorder and the tuning of magnetism in MnBi_2*n*_Te_3*n*+1_. Such understanding will provide fertile ground for designing schemes to realize emergent topological phenomena in this family of materials. The studies can also serve as guidance for future vdW topological magnetic realization based on the key structural ingredients.

## THE GROWTH OF HIGH-QUALITY M$\bf{n}$B$\bf{i}$_2$\boldsymbol{n}$_T$\bf{e}$_3$\boldsymbol{n}$+1_ SINGLE CRYSTALS

MnBi_2*n*_Te_3*n*+1_ is made of alternating SL and QL building blocks, which are depicted in Fig. 1(a). The SL (purple block) consists of a layer of closely packed MnTe_6_ octahedra sandwiched between two layers of BiTe_6_ octahedra, and they are connected by edge sharing. Each QL (blue block) can be seen as two layers of edge-sharing BiTe_6_ octahedra stacked along the *c* axis. MnBi_2*n*_Te_3*n*+1_ with *n* = 1 to *n* = ∞ can be constructed with these SL and QL ‘lego pieces’. The detailed crystal structural stacking rules of MnBi_2*n*_Te_3*n*+1_ are elaborated in [30,34].

MnBi_2*n*_Te_3*n*+1_ phases can be considered as quasi-metastable. Thus, high-quality single-crystal growth is challenging. The synthesis of MnBi_2*n*_Te_3*n*+1_ dates back to the very first discovery of MnBi_2_Te_4_ in 2013 [12]. Single-crystal growth of MnBi_2_Te_4_ became extensively studied when it was proposed as an intrinsic MTI in 2018. Today there are mainly three methods of growth to obtain MnBi_2_Te_4_ single crystals: congruent/incongruent melting, the flux method and the chemical vapor transport method. High-*n* members (*n* ≥ 2) in the MnBi_2*n*_Te_3*n*+1_ series can also be grown via similar growth methods, but with much more stringent growth conditions, especially the temperature control. It is noted that sizable phase-pure single crystals have only been made for *n* ≤ 4. We next go over each growth technique and discuss the pros and cons as a comparison.

### Growth from congruent/incongruent melting

The growth from the melt is done by cooling the stoichiometric melt of the targeted phase through its melting temperature [3,4,13,16,32,35,57,59,60]. Modified Bridgman and vertical Bridgman methods were used. According to the quasi-binary MnTe-Bi_2_Te_3_ phase diagram [13], MnBi_2*n*_Te_3*n*+1_ phases crystallize in an 8^○^ temperature window from the incongruent melt. Therefore, an as-grown ingot always has various *n* phases. Single-crystalline blocks of each phase can be isolated from the as-grown ingot. However, due to the intergrowth, especially on the high-*n* side, sizable pure-phase single crystals with *n* ≥ 4 have not been obtained even using the vertical Bridgman method, which can achieve more precise and consistent temperature control [60]. The mixture is typically heated to around 900 ^○^C, where they form a homogeneous liquid. Then it is quickly cooled to 600 ^○^C, where the mixture is still MnTe and Bi_2_Te_3_. Below 600 ^○^C, the [MnBi_2_Te_4_] SL structure starts to be more thermodynamically stable than MnTe, and MnBi_2*n*_Te_3*n*+1_ begins to form. To ensure that the process is complete, the mixture needs to be cooled slowly through its melting temperature. It was found that cooling faster than 0.5 ^○^C/h between 600 ^○^C and the growth temperature results in excessive Bi_2_Te_3_ and MnTe [57]. A slow cooling through the solid-liquid phase boundary and prolonged annealing just below the melting temperature are needed, to ensure that the final cooling or the dwelling can last weeks to a month.

In the vertical Bridgman growth, single crystals are grown by gradually moving the stoichiometric melt from the hot zone (∼677 ^○^C) to the cold zone (∼527 ^○^C) at a rate of 0.5 mm/h to cross the melt-solid interface. It requires a larger amount of growth material, but helps to achieve better temperature control in the synthesis. Via this method, despite unavoidable intergrowth for high-*n* members, one can obtain phases from MnBi_2_Te_4_ up to MnBi_14_Te_22_ (*n* = 7) from the stoichiometric melt [60]. One can also add MnCl_2_ into the growth mixture to provide an Mn-rich environment. Zero-field QAHE is observed at 1.4 K in one piece of MnBi_2_Te_4_ that was grown with additional MnCl_2_ [4].

### Growth from the flux method

Flux growth is the method to obtain single crystals out of a high-temperature solution, that is, a flux [61]. Upon cooling, when the solubility of the targeted phase in the flux becomes smaller than the actual concentration, the system becomes over-saturated, and single crystals start to precipitate to lower the concentration in the solution. The crystals nucleate and then continue to grow upon further cooling. For MnBi_2*n*_Te_3*n*+1_ growth, Bi_2_Te_3_ was used as the self-flux, or the solute [15]. Using this growth method, the plate-like single crystals obtained in each batch can be of single phase and sizable phase-pure single crystals have been obtained up to *n* = 4. In the growth, Mn, Bi and Te [15,28–30] are mixed at a ratio of MnTe : Bi_2_Te_3_ = *x* : 100 − *x* with 10 < *x* < 20. For high-*n* MnBi_2*n*_Te_3*n*+1_, a lower *x* is desired to ensure that there is enough flux for the growth. The mixture is placed in an alumina crucible and sealed under a vacuum inside a quartz tube. The ampule was initially heated to 900 ^○^C and held at that temperature for a few hours to ensure a homogeneous liquid. At this stage, the mixture can be considered as MnTe dissolved in Bi_2_Te_3_ liquid solution. This is followed by a quick cooling to around 600 ^○^C and then a slow cooling down to the targeted decanting temperature *T*_decant_ (see Table 1 below) in days to weeks. The nucleation rate of MnBi_2_Te_4_ depends on *x* and the cooling rate. In general, a smaller *x* and a slower cooling rate allow for fewer nucleation sites and lead to larger single crystals. After dwelling at *T*_decant_ for another few days, millimeter-to-centimeter-sized plate-like single crystals can be separated from the liquid flux via a centrifuge.

**Table 1.** Summary of the chemical, structural and magnetic properties of flux-grown MnBi_2*n*_Te_3*n*+1_ from *n* = 1 to 4. Here *SJ*_1_ and *SJ*_2_ refer to the NN and the NNN intraplanar interactions; *SJ_c_* is the NN interplanar interaction; *SD* is the uniaxial anisotropy energy. The data from high-energy inelastic neutron scattering (INS) measurement are fitted with the *J*_1_−*J*_2_ model. The data from *M*(H) are calculated using Equations(2)–(5) below.

	MnBi_2_Te_4_	MnBi_4_Te_7_	MnBi_6_Te_10_	MnBi_8_Te_13_
*n*	1	2	3	4
Space group	*R*-3*m*	*Pc*-3*c*	*R*-3*m*	*R*-3*m*
Magnetic space group	*R_I_*-3*c*	*Pc*-3*c*1	R_*I*_-3*c*	*R*-3*m*′
Lattice constant *a* (Å)	4.3336(2) [25]	4.3453(5) [25]	4.361 [34]	4.3749(1) [30]
Lattice constant *c* (Å)	40.926(3) [25]	23.705(3) [25]	101.300 [34]	132.415(3) [30]
d_*Mn* − *Mn*_ (Å)	13.642(1)	23.705(3)	33.995(1)	44.138(1)
Mn : Bi : Te (WDS)	0.90 : 2.11 : 4	0.79(2) : 4.29(8) : 7	0.79(1) : 6.30(2) : 10	0.74(3) : 8.2(1) : 13
*T*_decant_ (^○^C)	587	585	583	582
Magnetism	AFM	AFM	AFM	FM
*T_N_*/*T_C_* (K)	24	13	11	10.5
H$_{sat}^c$ (T)	7.8	0.22	0.2	0.12
H$_{sat}^{ab}$ (T)	11	1.2	1.2	1.2
*SJ_c_* (meV/Mn) from INS	−0.055 [23]	–	–	–
*SJ*_1_ (meV/Mn) from INS	0.31 [23]	–	–	–
*SJ*_2_ (meV/Mn) from INS	−0.06 [23]	–	–	–
*SD* (meV/Mn) from INS	0.12 [23]	–	–	–
*SJ_c_* (meV/Mn) from *M*(H)	−0.09 [65,66]	0.0086 [36]	0.0031 [36]	–
*SD* (meV/Mn) from *M*(H)	0.08 [65,66]	0.098 [36]	0.098 [36]	–
		0.052 [51,74]		

The key to successful MnBi_2*n*_Te_3*n*+1_ flux growth lies in temperature control. The growth is limited by the few-degree temperature window above *T*_decant_ where the targeted phase is stable. Therefore, delicate control of the final dwelling temperature *T*_decant_ is critical. A one-degree offset or less in *T*_decant_ may result in a different MnBi_2*n*_Te_3*n*+1_ phase. Given the temperature sensitivity, careful calibration is needed to determine and access *T*_decant_ consistently in a box furnace. Usually, a series of test growth runs needs to be done to determine the best temperature profile when a new furnace is in use. This test procedure has been detailed in [30]. In addition to the temperature control by the furnace, one should also keep track of other factors that can cause slight shifts in temperatures, such as the location of the growth ampule in the furnace, the relative vertical position of the material in the growth ampule and the length of the growth ampule to make sure that *T*_decant_ is repeatedly accessible.

Because *T*_decant_ is close to the melting point of the flux, a quick spin-out process (less than 5 s) is essential to remove the flux while it remains in its liquid form. Additional means can be taken to delay the solidification of the liquid flux. These methods include increasing the quantity of starting materials, preheating the ampule container of the centrifuge and incorporating extra layers of thermal insulation for the quartz ampule. For instance, applying the silver paste on the quartz walls or enclosing the growth ampule within a larger-diameter quartz tube can help enhance the insulating properties of the ampule.

Lastly, in flux growth, the flux is often embedded inside the targeted crystals. Indeed, unavoidable Bi_2_Te_3_-phase impurities can be found inside the targeted MnBi_2*n*_Te_3*n*+1_ phase grown by the flux method. X-ray diffractions (XRDs) can be used to carefully screen out the pieces with minimal embedded flux to ensure accurate measurements of their physical properties.

### Growth from the chemical vapor transport method

Chemical vapor transport (CVT) is a method that has been widely adopted to grow single crystals of vdW materials such as transition-metal chalcogenides. So far, single-crystal MnBi_2_Te_4_ and MnBi_4_Te_7_ can be grown this way. The growth involves a gaseous transport agent that can form intermediate volatile compounds with the constituting elements of the targeted material at the source end. Then at the sink side, which is the cold end for most reactions, the intermediate gaseous phases dissociate, condensing into the targeted phase and forming single crystals over time. For the growth of the MnBi_2*n*_Te_3*n*+1_ family, I_2_, TeI_4_ and BiI_3_ have been used as the transport agent [58,62]. In all cases, MnI_2_ becomes the dominant iodide species during the growth, and the concentration of the intermediate gaseous phase is limited by the volatility of MnI_2_, so there is no qualitative difference between these transport agents [62]. Alternatively, one can use chlorides such as MnCl_2_, TeCl_4_ and MoCl_5_ as transport agents. However, the transport with chloride is less efficient because MnCl_2_ and other intermediate chloride phases are less volatile than their iodide counterparts, so the transport rate is limited by the lower vapor pressure of MnCl_2_, resulting in smaller crystals [62].

Given the metastability of the MnBi_2*n*_Te_3*n*+1_ phase, it is unsurprising that MnBi_2*n*_Te_3*n*+1_ growth with vapor requires delicate control of the temperature too. Considerations are as follows. Firstly, the sink-end temperature should be well tested. The maximum temperature of the cold end should not exceed the melting point of MnBi_2*n*_Te_3*n*+1_, or the condensed phase will be in liquid form; moreover, at a higher temperature near 600 ^○^C, MnBi_2_Te_4_ is no longer stable and thus Bi_2_Te_3_ droplets and MnTe single crystals will appear. Secondly, if the cold-end temperature is too low, the growth will be limited by an insufficient Mn transport rate, so only Bi_2_Te_3_ forms. So, if a near-stoichiometric mixture is used as the source material, the cold-end temperature can be chosen to be *T*_decant_ [58]. But, if it is in the Mn-rich condition, the MnBi_2_Te_4_ phase can form over a range of cold-end temperatures and suppress the formation of high-*n* members of MnBi_2*n*_Te_3*n*+1_ as well as Mn-doped Bi_2_Te_3_ [62]. Thirdly, the temperature gradient should be small, in the range 3–20 K so that the cold-end temperature is more stable and the transport rate is not too high.

The CVT growth of MnBi_2*n*_Te_3*n*+1_ can be done in a box furnace, where the temperature is overall uniform and well controlled with a small intrinsic temperature gradient either vertically from top to bottom, horizontally from side to middle or from the middle to the furnace door. This is sufficient to drive transport growth. When a tube furnace is used, a careful calibration of the temperature at the hot and cold ends is necessary to ensure that the temperature condition is met to obtain the targeted phase.

A representative image from each growth is included in Fig. 1(c). The CVT-grown samples are typically millimeter sized and marked with well-defined hexagonal edges, (001) and (012) planes. In comparison, the crystal is less well defined when grown from the stoichiometric melt. The samples grown with flux are usually large in size with as-grown *ab* planes, but their edges are not well defined. Therefore, the unique growth habit of CVT-grown samples provides opportunities for in-plane and side surface measurements. In addition, the CVT crystals also tend to be Bi_2_Te_3_-free and Mn richer than flux growth for both MnBi_2_Te_4_ [58] and MnSb_2_Te_4_ [62], which may be beneficial for QAHE realization, as we discuss in the section entitled ‘Disorder: antisites and optimization’.

### Growth of chemically doped MnBi_2*n*_Te_3*n*+1_

Single-crystal series with Sb doping on the Bi sites, or Sn and Pb doping on the Mn sites have been made. There is no Se doping on the Te sites reported. The difficulty in obtaining the bulk form with Se doping stems from the fact that bulk MnBi_2_Se_4_ is only reported in the monoclinic (*C*2/*m*) non-vdW phase and only thin-film MnBi_2_Se_4_ grown by MBE shares the same structure as MnBi_2_Te_4_ [54].

The three growth methods for MnBi_2*n*_Te_3*n*+1_ can all be generalized to their isostructural non-magnetic sister compounds (Sn, Pb) (Bi, Sb)_2*n*_Te_3*n*+1_, and the chemically doped systems that exist continuously in between. In the flux growth of the Sb-doping series, since the melting point of Sb_2_Te_3_ is ∼35^○^ higher than that of Bi_2_Te_3_ [63], the decanting temperature must be correspondingly increased to be slightly higher than the melting point of (Bi_1−*x*_Sb_*x*_)_2_Te_3_, which increases with *x*. As a result, the optimal growth window is reduced. It was also found that the actual doping levels are slightly higher in flux growth than the nominal ratio [51].

When growing (Mn/X)Bi_2*n*_Te_3*n*+1_ by flux (X = Sn, Pb), adjustment of the synthesis condition is needed depending on the shift of the eutectic point between the (Mn/X)Bi_2*n*_Te_3*n*+1_ and Bi_2_Te_3_ fluxes. For flux growth of (Mn_1 − *x*_Sn_*x*_)Bi_2_Te_4_, the (Mn_1 − *x*_Sn_*x*_)Te : Bi_2_Te_3_ ratio and temperature profile remain the same for *x* up to 66% [64]. However, for flux growth of the Pb-doping series, although the temperature profile remains the same, a higher (Mn_1 − *x*_Pb_*x*_)Te : Bi_2_Te_3_ ratio is found to be critical for the successful flux growth when higher *x* is involved [65], likely because PbTe dissolves more easily in Bi_2_Te_3_ than MnTe and SnTe. In such a case, a series of test growth runs is necessary to locate the eutectic point and determine the optimal ratio and temperatures.

## EVOLUTION OF MAGNETISM IN M$\bf{n}$B$\bf{i}$_2$\boldsymbol{n}$_T$\bf{e}$_3$\boldsymbol{n}$+1_

**Figure 2.**
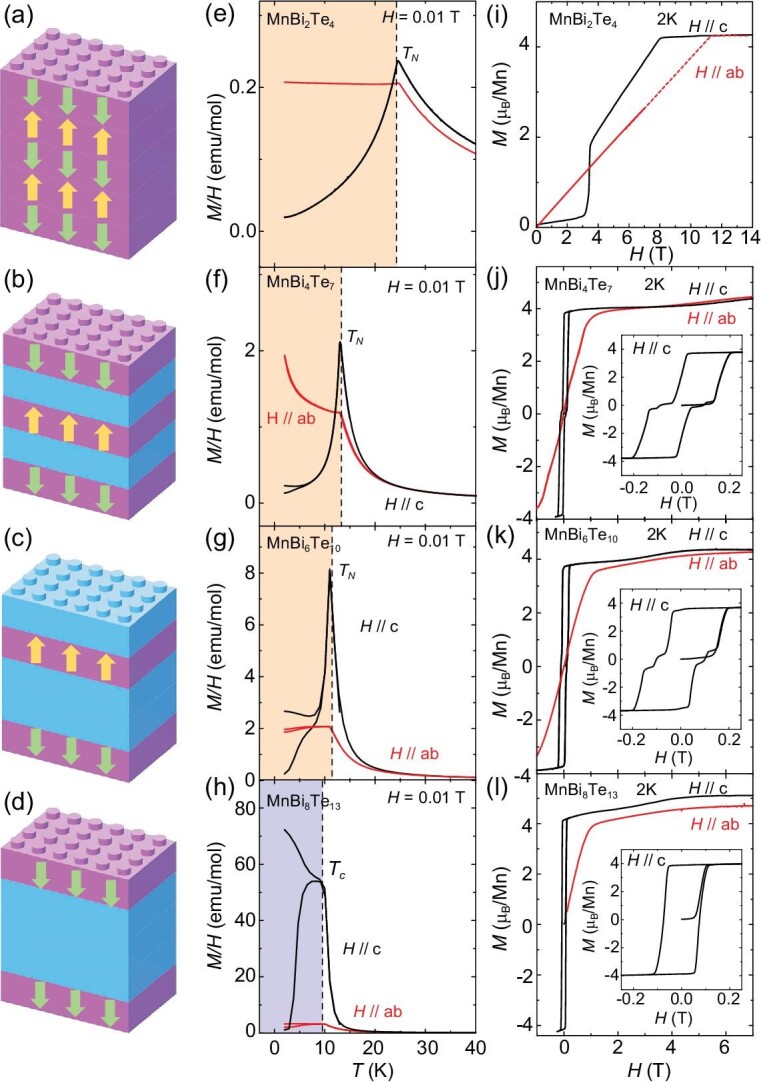
Magnetism of MnBi_2*n*_Te_3*n*+1_. (a–d) Magnetic structure for MnBi_2_Te_4_, MnBi_4_Te_7_, MnBi_6_Te_10_ and MnBi_8_Te_13_, respectively. (e–h) The temperature-dependent magnetic susceptibility measured with in-plane and out-of-plane magnetic fields. (i–l) Isothermal magnetization under in-plane and out-of-plane fields at 2 K. Insets of (j–l): the magnetization hysteresis loop near zero field. Figures are reproduced from [20,28,30,58].

MnBi_2*n*_Te_3*n*+1_ compounds with Mn^2 +^ (*S* = 5/2) ions offer an excellent and tunable platform for studying vdW magnetism since they all share the same building block for magnetism—the Mn layer in the middle of the SL, with varying interlayer coupling strengths. Table 1 summarizes the key parameters of their magnetic order and interactions. As shown in Fig. 2(a)–(d), within each SL, the FM Mn-Te-Mn superexchange interactions overwhelmingly win the AFM-preferred Mn-Mn direct interactions and result in an in-plane FM configuration. In MnBi_2_Te_4_, the interlayer coupling is AFM, which results in the A-type easy-axis antiferromagnetic structure. By increasing the number of Bi_2_Te_3_ spacer layers, the spacing between Mn-Mn layers and the superexchange path between them are effectively increased. Therefore, the AFM coupling is weakened and eventually becomes weaker than other mechanisms for FM coupling such as dipolar interactions, and antisite-mediated exchange, so the magnetism becomes FM along the *c* axis, as demonstrated by the magnetic properties summarized in Fig. 2(e)–(h). For those with an A-type AFM, when an external magnetic field *H* is applied along the easy *c* axis, a spin-flop transition appears in MnBi_2_Te_4_, and spin-flip transitions are observed in MnBi_4_Te_7_ and MnBi_6_Te_10_, as summarized in Fig. 2(i)–(l).

Such evolution of magnetism under an external magnetic field can be captured by the Hamiltonian for the magnetic ground state, as shown in


(1) \begin{eqnarray*}
E &=& -\sum _{ii^{\prime }}J_1{{\bf S_i}\cdot {S_{i^{\prime }}}}-\sum _{ii^{\prime }}J_2{{\bf S_i}\cdot {S_{i^{\prime }}}} -\sum _{ij}J_c{{\bf S_i}\cdot {S_j}} \\
&& -\sum _{i}D{S_{iz}}^2-\sum _{i} g\mu _B{\bf H}\cdot {\bf S}_{i,z}.
\end{eqnarray*}

The terms include strong FM intraplanar exchange coupling (dominated by *J*_1_ and *J*_2_ for the nearest neighbor (NN) and next-nearest neighbor (NNN), respectively), weak NN Mn-Mn interplanar coupling *J_c_*, magnetic anisotropy *D* that can generally be absorbed into the single-site term and Zeeman energy when an external magnetic field is present. Here, interactions beyond the NNN are omitted. We denote by *i, i*′ sites in the same SL, and by *i, j* sites in the neighboring SL; *D* > 0 is for our 2D easy-axis system, while *D* < 0 stands for the easy-plane anisotropy.

The evolution of magnetism results from the competition between the *J_c_* term, *D* term and the Zeeman term. Equation (1) can thereby be simplified into the bilayer Stoner–Wohlfarth model by only considering these three terms. By this [23,65,66], *SD* and *SJ_c_* can be estimated as


(2) \begin{eqnarray*}
|SD| & =(H_{sf}^{2}/H_{s}^{//c})(g\mu _{B}/2),
\end{eqnarray*}

(3) \begin{eqnarray*}
|SJ_{c}| =(g\mu _{B}/2z)(H_{sf}^{2}/H_{s}^{c}+H_{s}^{ c}).
\end{eqnarray*}

Here ${z}$ is the number of NN Mn of an Mn ion, which is 6 for *n* = 1, 3, 4 and 2 for *n* = 2. We denote by *H_sf_* the spin-flop field and by $H_{s}^{//c}$ the saturation field with *H*//*c*. We can also obtain *SD* and *SJ_c_* by


(4) \begin{eqnarray*}
|SD| & =(H_{s}^{//ab}-H_{s}^{//c})(g\mu _{B}/4),
\end{eqnarray*}

(5) \begin{eqnarray*}
|SJ_{c}| & =(H_{s}^{//ab}+H_{s}^{//c})(g\mu _{B}/4z).
\end{eqnarray*}

Here $H_{s}^{//ab}$ denotes the saturation field with *H*//*ab*. The spin gap can be estimated as $\Delta =2SD\sqrt{zSJ_c/SD+1}$.

By such, experimental estimations of *SJ_c_*, and *SD* from the *M*(H) data for MnBi_2*n*_Te_3*n*+1_ (*n* ≤ 3) are summarized in Table 1. Meanwhile, *SJ_c_, SJ*_1_, *SJ*_2_ and *SD* determined from inelastic neutron scattering (INS) are also summarized in Table 1. The values obtained from these two methods agree with each other in magnitude. Among all MnBi_2*n*_Te_3*n*+1_, MnBi_2_Te_4_ has the strongest interlayer exchange, and the ratio of |*J_c_*/*J*_1_| measured by INS is ∼0.18 [23]. The ratio is expected to be much lower for other MnBi_2*n*_Te_3*n*+1_ that have much smaller |*J_c_*| but comparable |*J*_1_| within the SL. The small |*J_c_*/*J*_1_| is unsurprising considering their long interlayer Mn-Mn superexchange path, which ranges from 13.642 Å in MnBi_2_Te_4_ to 44.1 Å in MnBi_8_Te_13_. This leads to a much weaker interlayer exchange interaction than the intralayer ones and thus quasi-2D magnetism.

From *n* = 1 to 3, *J* and *D* are generally of the same order of magnitude in SL, while *J_c_* decreases the most and affects the magnetic behavior the most. Firstly, a 2D easy-axis system with *D* > 0 orders at a general temperature of


(6) \begin{eqnarray*}
T_{c}=\frac{a|J|}{b+\log (|J/J_{\rm {eff}}|)},
\end{eqnarray*}

where *a* and *b* are constants of the order of 1, and *J*_eff_ is a combination of *J_c_* and *D* that reduces to *D* in the *J_c_* → 0 limit [67]. Since MnBi_2_Te_4_ has the strongest *J_c_* and thus *J*_eff_, according to Equation (6), it has the highest *T_c_* of 24 K. The temperature *T_c_* quickly decreases to 13 K in MnBi_4_Te_7_ and then slowly decreases to 10.5 K in MnBi_8_Te_13_ since *J*_eff_ reduces to ∼*D* in these high-*n* members. Secondly, in MnBi_2_Te_4_, the ratio of |*J_c_*/*D*| ∼ 0.45 suggests the comparable *J_c_* and *D* here and thus a weak anisotropy scenario. The competition between *J_c_, D* and the Zeeman term leads to a spin-flop transition as the three terms prefer the spins in opposite configurations, along the easy *c* axis and along the field direction, respectively. Together, a canted AFM state that minimizes the overall energy is favored in the intermediate field regime [17]. On the other hand, in MnBi_4_Te_7_ and MnBi_6_Te_10_ where |*D*/*J_c_*| is much larger, the system exhibits strong anisotropy and thus undergoes a spin-flip transition. Thirdly, as a result of smaller *J_c_*, the saturation field in MnBi_4_Te_7_ and MnBi_6_Te_10_ is 40 times smaller than that in MnBi_2_Te_4_. The minor step at 0.1 T for MnBi_6_Te_10_ is possibly due to some FM domain formations and it has been universally observed in MnBi_6_Te_10_ [29,32,36]. Lastly, with *H*//*ab*, the much reduced *J_c_* in high-*n* members also leads to a 10 times smaller saturation field of 1.2 T than 11 T in MnBi_2_Te_4_.

Eventually, at *n* = 4, MnBi_2*n*_Te_3*n*+1_ becomes an axion ferromagnet that shows long-range FM ordering at 10.5 K with clear FM hysteresis at 2 K. Relaxation behaviors are observed. One scenario to understand this relaxation is to look at MnBi_8_Te_13_ as a truly 2D magnet with independent FM planes, the so-called ‘single-layer magnetism’, a 2D planar analog of the more commonly known single-molecule magnet [68], which should give rise to spin-glass-like magnetic relaxation behavior. An alternative scenario is the conventional ferromagnet picture that attributes the relaxation behavior to the irreversible FM domain movements [69]. Indeed, relaxation behavior was also reported in the AC susceptibility in Sb-doped MnBi_4_Te_7_ and MnBi_6_Te_10_ as well as in ferromagnetic MnSb_2_Te_4_ [68–71]. Furthermore, the vanishing interlayer magnetic interactions lead to the low formation energy of the magnetic domain boundary and thus very soft FM domains here. As a result, rare ‘double-peak’ behavior is observed in the AC susceptibility under small DC bias fields [69].

It is worth noting that, for MnBi_4_Te_7_ and MnBi_6_Te_10_ with different growth methods or growth conditions, FM ground states were also observed [70,72]. The disparities are likely attributed to varying degrees of crystal defects within samples grown by different groups, which we discuss extensively in the next section.

Another important feature of magnetism in MnBi_2*n*_Te_3*n*+1_ is the robust in-plane FM fluctuations that tend to persist above the ordering temperatures and may induce the Zeeman gap for the QAHE realization [73]. High-frequency or high-field electron spin resonance (HF-ESR) spectroscopy reveals a zero-field FM excitation gap of 0.11 meV up to 30 K in MnBi_4_Te_7_ [74]. Although the zero-field AFM gap of 0.45 meV in MnBi_2_Te_4_ closes at *T_N_*, HF ESR finds FM-type spin fluctuations above *T_N_* in MnBi_2_Te_4_ at magnetic fields above 6 T. Hence, it is the generic property of the SLs that short-range FM spin correlations persist far above the ordering temperature, provided that the SLs are decoupled either by spacer layers or moderate magnetic fields. Spatially varied magnetic fluctuations were also imaged by nitrogen-vacancy relaxometry in MnBi_4_Te_7_ nanoflakes above its ordering temperature [75].

## DISORDER: ANTISITES AND OPTIMIZATION

Although the Chern insulator state can be observed in the polarized FM state of MnBi_2_Te_4_ under high magnetic fields even above *T_N_* = 24 K [6], the zero-field QAHE was only reported below the critical temperature *T*_QAHE_ = 1.4 K in a five-layer device [4]. At zero field, the magnetic inhomogeneity arising from the chemical disorder reduces the universal magnetism-induced surface gap. This reduction in the gap prevents the achievement of the QAHE at higher temperatures. Therefore, to have a high success rate in realizing QAHE and an enhanced ratio of *T*_QAHE_/*T_N_* in an MnBi_2_Te_4_ device, understanding and controlling its defects is essential.

The typical defects in MnBi_2*n*_Te_3*n*+1_ include antisite defects, vacancies and stacking faults [76]. They have played an important role in the magnetism and band topology in the MnBi_2*n*_Te_3*n*+1_ family. For example, in the case of MnBi_6_Te_10_, an FM state can be achieved by having additional Mn vacancies in the sample [72]. In addition to these defects in the bulk samples, when the crystals are exfoliated and fabricated into devices, the fabrication process can introduce defects too. For example, the synergistic interaction between the Mn-Bi antisite defects and Te deficiency induced by traces of oxygen in the fabrication environment may promote surface reconstruction [77]. In this review, we focus only on the defects in bulk crystals.

Scanning transmission electron microscopy (STEM), STM, X-ray and neutron diffractions, and magnetization analysis have identified and quantified the chemical defects in MnBi_2*n*_Te_3*n*+1_ [15,16,23,25,34,51,58,72,78–84]. Each technique has its pros and cons. For example, STEM and STM can directly visualize the defects, but quantitative analysis of defect concentration is hard with STEM because the image is an average of the overall slab, and STM is only surface sensitive; diffraction techniques are bulk measurements, but the refined results depend on the structural model used; the magnetization analysis is only sensitive to the magnetic elements and requires high magnetic fields to polarize the spins. Therefore, as complementary techniques, it is beneficial to compare the results of these techniques. The topographic STM imaging directly visualizes the antisite defects on the cleaved (001) surface [15,78]. It reveals predominant Mn_Bi_ (Mn substitution of Bi atoms on the Bi site) and minor Bi_Te_ (Bi substitution of Te atoms on the Te site) antisite disorders. Bi_Te_ makes up around 0.2% of Te sites in MnBi_2_Te_4_, while Mn_Bi_ accounts for 3% of Bi sites in flux-grown MnBi_2_Te_4_ crystals [15,78] and 5% in congruent-melting-grown MnBi_2_Te_4_ [79]. This is not surprising since compared to congruent-melting growth, the flux growth contains stoichiometrically less Mn in the starting materials. Similar defect patterns were commonly observed in the STM images of Mn-doped Bi_2_Te_3_, and also in the QL of MnBi_4_Te_7_ [80,81]. For MnBi_4_Te_7_, the amount of Mn_Bi_ is suggested to be 2.1% in the SL and 3% in the QL [80].

XRD or neutron diffraction provides an indirect route to obtain defect concentrations, which may depend on the structural model used for the refinement. Since the neutron scattering lengths of Mn and Bi have opposite signs, neutron diffraction is unique in resolving the occupancy of Mn and Bi on each site. In MnBi_2_Te_4_ grown via the congruent-melting growth, XRD refinement was performed [16,79]. The refinement was carried out using a charge neutrality constraint and a structural model that allowed for Mn-Bi antisite disorder and Mn vacancies, while Te was assumed to occupy full occupancy on Te sites. The results suggest that 21.5(1)% Bi_Mn_, 5.7(1)% Mn_Bi_ and 4.9(1)% of Mn sites are voids [16], resulting in Mn_0.85(3)_Bi_2.10(3)_Te_4_. If the vacancies are not included in the refinement, the percentages for Bi_Mn_ and Mn_Bi_ become 17.5% and 8.8% [79], which will lead to a stoichiometry inconsistent with the chemical analysis. In a flux-grown MnBi_2_Te_4_ studied by single-crystal neutron scattering that assumes full occupancy of Te, 18(1)% of Mn is occupied by Bi in MnBi_2_Te_4_ and only a negligible amount of 1(1)% Bi sites are occupied by Mn [25], leading to Mn_0.84_Bi_2.16_Te_4_. The single-crystal neutron measurement also suggests that the Bi_Mn_ percentages are 27.9(4)%, 34(1)% and 36(4)% for MnBi_4_Te_7_, MnBi_6_Te_10_ and MnBi_8_Te_13_ respectively, with an amount of Mn_Bi_ that is below the measurement resolution of neutron scattering [25,34].

**Figure 3.**
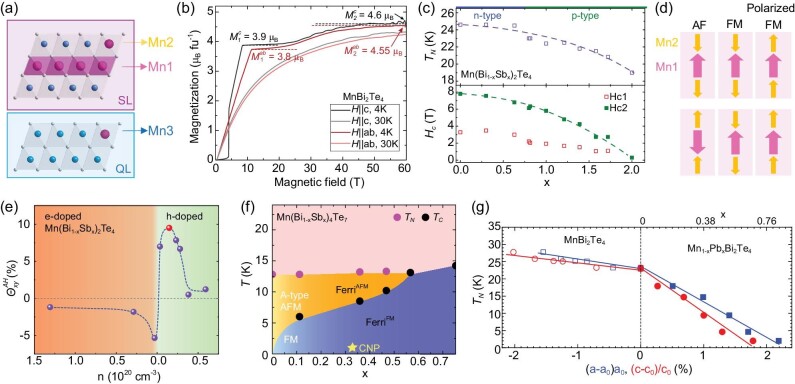
Antisite defects and the effect of Sb doping in MnBi_2_Te_4_ and MnBi_4_Te_7_. (a) Mn1 (Mn_Mn_), Mn2 (Mn_Bi_ in the ([MnBi_2_Te_4_] SL) and Mn3 (Mn_Bi_ in the [Bi_2_Te_3_] QL). (b) Isothermal magnetization *M*(H) in MnBi_2_Te_4_ up to 60 T for *H*‖*c* and *H*‖*ab* [83]. (c) The doping dependence of the ordering temperature *T_N_*-*x* and saturation fields *H_c_*-*x* of Mn(Bi_1 − *x*_Sb_*x*_)_2_Te_4_ grown by flux [66]. (d) The evolution of the magnetic structure of Mn(Bi_1 − *x*_Sb_*x*_)_2_Te_4_ under high field. AFM represents the magnetism of an AFM-like ground state. FM represents the magnetism of an FM-like ground state. Polarized FM represents the magnetism when all Mn moments are polarized at 50 T [83]. (e) The anomalous Hall conductivity with carrier concentration [48] in FM MnBi_2_Te_4_, consistent with the proposed type-II Weyl nodes. (f) The *T*−*x* phase diagram of Mn(Bi_1 − *x*_Sb_*x*_)_4_Te_7_ [51]. (g) Temperature *T_N_* versus the lattice parameter change in MnBi_2_Te_4_ under external pressure [87] and Pb doping [65].

Isothermal magnetization analysis provides one more way to estimate the small amount of Mn antisites. In [83], a method for estimating the antisite concentration was developed for MnBi_2_Te_4_ based on knowledge of the magnetic structure and its evolution under magnetic fields. Here, to better distinguish the Mn sites, we denote the Mn_Mn_ as Mn1 and the Mn_Bi_ as Mn2, as shown in Fig. 3(a). Here Mn1 concentrations are affected by Mn_Bi_ as well as Mn vacancies. In MnBi_2_Te_4_, each Mn sublattice is an A-type AFM, while Mn2 spins are strongly AFM coupled to Mn1 spins. Lai *et al.* [83] found that in the *M*(H) curve of MnBi_2_Te_4_, besides the spin-flop transition and saturation at 7.8 T, as shown in Fig. 2(i), one more metamagnetic transition was observed with a plateau around 50 T, as shown in Fig. 3(b). The intriguing *M*(H) can be understood as follows. At 7.8 T, an individual Mn1 or Mn2 sublattice enters their polarized FM state, while Mn1 and Mn2 are still strongly AFM coupled; it takes 50 T to make Mn2 spins FM aligned with the Mn1 spins [83]. Therefore, *M*(7.8   T)∝*n*_Mn1_ − 2*n*_Mn2_ and *M*(50   T)∝*n*_Mn1_ + 2*n*_Mn2_. By this, *n*_Mn1_ and *n*_Mn2_, the respective concentrations of Mn1 and Mn2, are estimated to be 83.5% and 3.2% in the flux-grown sample, whereas in the CVT-grown sample, they are estimated to be 87.4% and 3.8% [58]. The method has also been used to estimate the antisite amount in MnBi_4_Te_7_ where there are more metamagnetic plateaus due to the additional Mn3 site (Mn_Bi_ in Bi_2_Te_3_ QL) [51], as shown in Fig. 3(a). By this method, the Mn1, Mn2 and Mn3 concentrations are estimated as 73%, 1.5% and 3% in MnBi_4_Te_7_ [51], which are consistent with the STM measurements [80].

While defect formation during crystal growth is entropy driven and therefore inevitable, there are also internal structural factors that further promote the dominant antisite defects. It is argued that the internal strain of the MnTe layer is responsible for the high density of Bi_Mn_ defects, and its low formation energy was confirmed by DFT [85]. Compared to that in pure MnTe with in-plane lattice constant *a* = 4.15 Å, the Mn layer in MnBi_2_Te_4_ is stretched to *a* = 4.31 Å. Given the small atomic size of Mn, it is energetically favorable to form Mn vacancies and for large Bi atoms to enter to relieve the strain in the Mn layer, resulting in the overall decrease of the Mn1 concentration. As *n* increases, the lattice parameter *a* of the MnBi_2_Te_4_ layer is brought closer to Bi_2_Te_3_ in order to form a stable natural heterostructure, resulting in *a* ∼ 4.37 Å in high-*n* MnBi_2*n*_Te_3*n*+1_, so more Bi_Mn_ have to enter to relieve the strain on the Mn layer. Indeed, via neutron diffraction, Bi_Mn_ is found to increase from 18(1)% in MnBi_2_Te_4_ to 36(4)% in MnBi_8_Te_13_ [25,34]. Meanwhile, an opposite compressive effect is present but milder in the Bi layer since *a* ∼ 4.40 Å in Bi_2_Te_3_, so Mn_Bi_ will happen too but at a lesser extent, varying from 1(1)% to 3% of the Bi sites in flux-grown MnBi_2_Te_4_ crystals [15,78] and 4-5% in congruent-melting and CVT-grown samples [58,79]. Thus, overall, MnBi_2_Te_4_ appears Mn deficient. The same is happening in high-*n* members with slightly smaller Mn_Bi_, as suggested by the analysis of the isothermal magnetization and neutron diffraction [51,72].

Since Bi_Mn_ and Mn_Bi_ are correlated negatively, we may only choose to optimize one or the other. For device application and the search for QAHE, it is important to understand the roles of Mn_Bi_ and Bi_Mn_. Mn1 provides the major source of magnetism in the material, so Bi_Mn_ results in inhomogeneous Zeeman fields and reduces the associated Dirac gap in the surface states. In addition, one Bi_Mn_ donates an additional electron to the system, which brings in the spatial variation of the Fermi level and further reduces the global surface state gap. On the other hand, Mn_Bi_ weakens the overall effective Zeeman field in the system and acts as an electron acceptor, and the effect is inhomogeneous too.

Ways to tune defects include varying growth conditions, growth methods and chemical doping. In general, slower cooling and additional thermal annealing are two common routes to ensure thermodynamic equilibrium during the growth and give more uniform single crystals. Meanwhile, reducing the antisite formations remains an active topic in the crystal growth of this family of compounds. Growth efforts have shown that, for the flux-grown and CVT-grown samples, the analysis of the isothermal magnetization data revealed that the Bi_Mn_ percentages are 16.5% (flux grown) and 12.6% (CVT grown) and that the Mn_Bi_ percentages are 3.2% (flux grown) and 3.8% (CVT grown) [58]. Therefore, growing crystals in an Mn-rich environment indeed increases the Mn occupancy on the Mn site that is good for the realization of the QAHE; this, however, also slightly increases Mn_Bi_, which is detrimental to the QAHE. Calculation of the defect formation energy also suggests that Mn_Bi_ can be minimized in a Te-rich condition [85]. For growth from melting, temperature control can be critical for affecting the overall Mn level: FM MnBi_6_Te_10_ is grown at a temperature 5^○^ higher than that for AFM MnBi_6_Te_10_. It is found that the FM sample contains more Mn vacancies than the AFM samples, and overall Mn levels are lower [72]. Chemical doping provides another route to change the free energy of formation for different defect types. For example, Sb substitution of Bi leads to more Mn_Bi_ antisites, which we discuss more extensively in the next section.

The defects are also nontrivial in the aspect of topology. In ARPES, there have been discrepant reports on the Dirac point being slightly gapped or gapless in the surface states of MnBi_2_Te_4_. It was shown that the AFM-coupled Mn2 antisites can greatly reduce the Dirac point gap size locally [56,86]. It is also found that CVT-grown MnBi_2_Te_4_, which has more Mn1 and Mn2, is favorable to consistently achieving a quantized Hall resistance at high field [58]. The underlying picture could be that Mn1 increases much more than Mn2, leading to an overall spatially more homogeneous magnetization. Therefore, increasing the total Mn in crystal growth may benefit the realization of quantized Hall resistance.

For high-*n* MnBi_2*n*_Te_3*n*+1_ systems, although the interlayer FM state becomes more accessible with a larger *n*, the amount of Mn1 also decreases, making the sample more electron doped. As a result, it is difficult to tune the device to the CNP and the device studies are so far much more limited.

## THE EFFECT OF CHEMICAL DOPING

The as-grown MnBi_2*n*_Te_3*n*+1_ is heavily electron doped. ARPES measurement shows that the CNP of MnBi_2*n*_Te_3*n*+1_ is located 0.24–0.4 eV below the Fermi level, much larger than the bulk or surface gap [20–22,24,28,30,45]. Therefore, the transport properties arising from the topological features are screened by the electron conduction from the bulk. This hinders access to the topological states and motivates studies to lower the Fermi energy through Sb doping. Since Sb has a relatively closer electronegativity and ionic radius to Mn compared to Bi, substitution of Bi with Sb significantly increases the amount of Mn_Bi/Sb_, which adds holes in the system and makes the charge carriers go from electron type to hole type. However, the additional magnetic sublattice arising from the increased Mn_Bi/Sb_ antisites also results in complex magnetism [19,51,66,70,88,89].

According to Yan *et al.* [66], Mn(Bi_1 − *x*_Sb_*x*_)_2_Te_4_ remains AFM for the whole doping range, *T_N_* and the spin-flop and saturation fields monotonically decrease with *x*, as presented in the *T*-*x* and *H*-*x* phase diagrams in Fig. 3(c). However, at higher *x*, drastically different magnetic ground states are found in different literature, with some showing FM behavior and some remaining AFM [66,82,90–94]. Neutron scattering measurement on one AFM and one FM MnSb_2_Te_4_ sample distinguished the underlying difference [82]. As shown in Fig. 3(d), in both samples, Mn1 and Mn2 sublattices are antiferromagnetically coupled to each other [82,90]. In the one that shows AFM behavior, an individual Mn1 or Mn2 sublattice is A-type AFM, while in the other one that shows FM behavior, an Mn1 or Mn2 sublattice is individually FM. This ferrimagnetic nature leads to a reduced magnetic moment at 7 T observed in experiments. The actual composition is determined to be Mn_0.588_Sb_0.412_(Sb_0.871_Mn_0.129_)_2_Te_4_ for the former and Mn_0.635_Sb_0.365_(Sb_0.850_Mn_0.150_)_2_Te_4_ for the latter, indicating the existence of a critical level of Mn2 that can switch the interlayer interaction of Mn1 from AFM to FM. In practice, the defect concentration can be delicately controlled with slightly different temperature profile, starting material from the growth [82] and growth method [62].

Sb doping adds holes to the system since Mn_Sb_ (electron acceptors) formation is more energetically favorable than (Sb)_Mn_ (electron donors) [85]. Indeed, the charge carrier density evolves linearly with doping and a cross-over from electron to hole behavior is found near *x* = 0.315 in MnBi_2_Te_4_ [19,66]. The trend was also directly identified via ARPES [19], where the Fermi level is tuned near the Dirac point around the nominal concentration of *x* = 0.3. The control of the chemical potential with Sb doping thus allows one to access and study the topological states by transport measurements. For example, a recent report shows that devices fabricated with flux-grown Mn(Bi_1 − *x*_Sb_*x*_)_2_Te_4_ single crystal have quantized Hall resistance above 6 T [95]. In particular, the effect is achieved near zero gating near the CNP. The capability of tuning the chemical potential via Sb doping also enables one to map the band structure via quantum oscillations to search for new topological states. In the bulk form, FM MnBi_2_Te_4_ is proposed to be a clean type-II Weyl semimetal [1,2]. Because the FM can only be achieved under high field, which limits the use of other band structure measurement techniques such as ARPES, quantum oscillation is the right tool here. One way quantum oscillation is commonly used for this purpose is by extracting the Berry phase from the Lifshitz-Kosevich fitting. However, because the system is intrinsically time-reversal-symmetry breaking, the extracted phase factor is not guaranteed to be π or 0 [96,97]. Given its clean band structure, one can in principle map out the exact evolution of electron and hole pockets near the CNP, and then compare it with the DFT calculations to try to confirm the type-II Weyl band structure [48,97]. The result is that the extracted frequency and effective mass from the quantum oscillation are consistent with the DFT calculation [97]. More recently, a signature intermediate state where both electron and hole pockets coexist has been reported by Jiang *et al.* [50], who provided direct evidence of the type-II Weyl nodes. Meanwhile, there is other transport evidence supporting the type-II Weyl semimetal state. One observation is the negative longitudinal magnetoresistance due to a chiral anomaly [48]. However, one needs to distinguish the effect of a chiral anomaly and suppressed magnetic scattering at high fields. The chiral anomaly occurs when E//B, so the negative magnetoresistance (MR) under the magnetic field caused by the chiral anomaly is strongly angular dependent, while negative MR induced by spin scattering is independent of the field direction. The recent angular-dependent measurements in [49] show that the negative MR in Sb-doped MnBi_2_Te_4_ is consistent with the chiral anomaly expected for the Weyl states. The other signature of type-II Weyl states is the sharp mobility peak and diverging anomalous Hall conductance that flips sign at the CNP, as shown in Fig. 3(e) [48,50,98], contrasting a type-I Weyl semimetal whose anomalous Hall conductance would remain constant across the CNP.

For MnBi_4_Te_7_ systems [51], the CNP is at *x* = 0.36. The switching of the magnetism on the Mn1 sublattice is continuously controlled in Mn(Bi_1 − *x*_Sb_*x*_)_4_Te_7_. Here, besides Mn1 and Mn2, an additional Mn3 (Mn_Bi_ in the QL) sublattice exists (see Fig. 3(a)). The interlayer exchange interaction of Mn1 is the result of the competition between the superexchange interaction bridged by the nonmagnetic Bi/Sb/Te anions and the exchange interaction mediated by Mn3. The former favors AFM here and the latter always favors FM. When Sb is doped, Mn1 decreases, while Mn2, Mn3 and total Mn concentrations increase [51]. Therefore, upon increasing *x*, the Mn3-assisted FM interaction becomes stronger and wins. Upon cooling, since the Mn3-assisted interaction follows the Curie-Weiss law and the superexchange interaction is weakly temperature dependent for a 2D system, FM interaction wins at lower temperatures. This scenario explains the *T*−*x* phase diagram shown in Fig. 3(f). By varying the growth condition, it is possible to obtain different antisite levels, leading to some seemingly controversial observations. For example, FM Mn1-Mn1 interlayer interaction can dominate even in pure MnBi_4_Te_7_ at low temperatures and lower Sb dopings, while AFM Mn1-Mn1 interlayer interaction can also appear in MnSb_4_Te_7_ [70,88,99]. Such delicate magnetic interaction in this system allows versatile means to easily trigger metamagnetic transitions, as we will see in the next section. Similarly, FM Mn1-Mn1 interaction can be achieved in Mn(Bi_1 − *x*_Sb_*x*_)_6_Te_10_ at even smaller doping [68,89].

When the Mn site is substituted by X (X = Sn/Pb) [64,65], the main effect is the dilution of the Mn sublattice. This effectively modifies the Hamiltonian of the energy scheme in (Mn_1 − *x*_X_*x*_)Bi_2_Te_4_ by introducing an additional factor of δ = 1 − *x* on the effective spin of the Mn/X site [65], leading to the linear decrease of *T_N_* with *x*, as shown in Fig. 3(g). X doping also changes the distribution of antisite defects. For example, the refinement of the neutron diffraction data on the (Mn_0.55(4)_Pb_0.33(4)_)Bi_2.10(2)_Te_4_ single crystal will not converge if Mn_Bi_ is present. In this case, the minimization of Mn_Bi_ could be a result of the Mn-lesser environment and an increase in lattice parameter *a* upon Pb doping. Consequently, the electron carrier density increases with doping. The reduction of Mn_Bi_ makes X doping a promising tuning parameter to optimize Mn1 and Mn2 in MnBi_2_Te_4_ crystals for the realization of QAHE.

## TUNING THE GROUND STATES BY EXTERNAL PRESSURE

**Figure 4.**
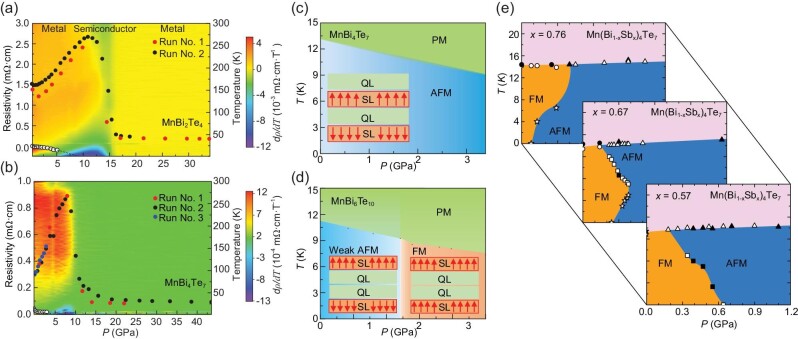
The temperature-pressure (*T*−*P*) phase diagrams of MnBi_2*n*_Te_3*n*+1_. (a and b) The *T*−*P* phase diagrams of MnBi_2_Te_4_ and MnBi_4_Te_7_, respectively, up to ∼40 GPa. Filled circles represent different runs of resistivity measurements at 1.8 K and open circles indicate AFM transition temperatures obtained from transport measurement [100]. (c and d) The *T*−*P* phase diagrams of MnBi_4_Te_7_ and MnBi_6_Te_10_ up to ∼3 GPa, respectively [101]. (e) The *T*−*P* phase diagrams of Mn(Bi_1 − *x*_Sb_*x*_)_4_Te_7_ below 1.2 GPa. Filled and open data points are obtained from transport and magnetization measurements, respectively [67].

External pressure offers a continuous tuning parameter of the system in a clean and non-intrusive way. By compressing the lattice and thereby modifying the interaction strength and band structure, it can serve as a powerful tool in tuning the ground state and topological properties. The high-pressure study has been carried out among MnBi_2*n*_Te_3*n*+1_ and its extended material family. Figure 4 summarizes a few selected pressure phase diagrams.

The pressure measurements of MnBi_2_Te_4_ were conducted up to tens of gigapascals [87,100]. As shown in Fig. 4(a), there is a complete suppression of *T_N_* at around 9 GPa. The resistivity at 1.8 K, ρ (1.8 K), first increases slightly with pressure, then undergoes a sharp drop around 15 GPa, where XRD suggests that it enters an amorphous state and the vdW gap vanishes [100]. MnBi_4_Te_7_ remains overall metallic in the transport measurement with a reduction in resistivity above 10 GPa in Fig. 4(b), and XRD suggests a structural transition to a monoclinic phase at 15.9 GPa and then a cubic phase at 18.6 GPa with no observation of an amorphous phase [100]. A closer examination below 3.5 GPa shows an almost linear decrease of *T_N_* [100,101] and an increase in the saturation field with pressure, shown in Fig. 4(c). DFT calculations suggest that, when external pressure compresses the lattice, it increases the interlayer exchange interaction, leading to the increased saturation field according to Equation (5), while it also weakens the intralayer FM coupling, leading to the decreased *T_N_* according to Equation (6) [101]. In MnBi_6_Te_10_ where the interlayer coupling is even weaker, both *T_N_* and the saturation field decrease with pressure (Fig. 4(d)), suggesting an accompanying decrease in the interlayer and intralayer couplings in this system. Surprisingly, when pressure exceeds 1.2 GPa, the overall Mn1-Mn1 interlayer interaction switches from AFM to FM. The authors argue that this is because the nearly vanishing interlayer AFM coupling is suppressed by the stronger FM intralayer fluctuations [101].

Pressure-driven metamagnetic transitions were observed in Mn(Bi_1 − *x*_Sb_*x*_)_4_Te_7_ [67]. Here, external pressure fine-tuned the already-delicate FM and AFM energy scales, resulting in distinct temperature-pressure phase diagrams for different *x* (Fig. 4(e)). The magnetic ordering temperature (upper phase line) remains little changed under pressure. According to Equation (6), the ordering temperature depends logarithmically weakly on the ratio *J*/*J*_eff_ and is thus weakly pressure dependent. On the other hand, the metamagnetic transition temperature *T_M_* phase line (the lower phase line) is unconventional. It is very sensitive to the pressure and temperature, and varies with doping: *dT_M_*/*dP* < 0 for *x* ≲ 0.6, *dT_M_*/*dP* > 0 for *x* ≳ 0.7; in particular, *dT_M_*/*dP* is positive at low temperatures and negative at higher temperatures for *x* = 0.67, showing an AFM-FM-AFM re-entrance behavior upon cooling. These unconventional lower phase lines arise from the competition between the superexchange Mn1-Mn1 AFM interaction and the Mn3-assisted FM Mn1-Mn1 interaction that we discussed in the previous section. Since the strength of the Mn3-assisted FM interaction is proportional to the susceptibility of Mn3, upon cooling, such a competition can result in an AFM-FM transition at small Mn3 concentrations, an AFM-FM-AFM transition at intermediate Mn3 concentrations and an FM-AFM transition at higher Mn3 concentrations. This leads to the observed *T*-*P* phase diagram across different doping regions [67].

The effect of pressure was measured in MnSb_2_Te_4_ [102] and MnSb_4_Te_7_ [103] up to 40 and 80 GPa, respectively. The temperature *T_N_* was suppressed under pressure and structural phase transitions were observed. MnSb_2_Te_4_ enters into an amorphous-like phase above 16.6 GPa, while MnSb_4_Te_7_ goes from trigonal *P*-3*m*1 to monoclinic *C*2/*m* at 16.2 GPa and then to a simple cubic *Im*-3*m* phase at 25.7 GPa. In particular, a superconducting state emerges at 30 GPa in MnSb_4_Te_7_ [103], which maxes out at 50.7 GPa with a critical temperature of 2.2 K. This is the first report of superconductivity in the series, but it appears in the high-pressure cubic phase instead of the vdW trigonal *P*-3*m*1 phase.

## OUTLOOKS

MnBi_2*n*_Te_3*n*+1_ and its extended family provide a material platform with excellent tunability in structure, magnetism and band topology. The compatibility between the non-magnetic QL and the magnetic SL leads to the MnBi_2*n*_Te_3*n*+1_ series where the magnetism evolves from a strongly interlayer coupled A-type AFM TI at *n* = 1, to a weakly interlayer coupled A-type AFM TI at *n* = 2, 3 and to an FM axion insulator at *n* ≥ 4. Moreover, magnetism in these systems can be fine-tuned through lattice modulations and defect redistribution. In the weak interlayer interaction limit especially, the magnetism of this material family can be manipulated by growth conditions, chemical doping and external pressure. Combined with the non-trivial topological properties of the materials, the versatile magnetism opens up the possibility of realizing emergent phenomena including QAHE, QSHE, etc.

To realize the exotic states predicted in MnBi_2*n*_Te_3*n*+1_, sustained efforts are needed to produce high-quality single crystals. As defects are inevitable, defect engineering is necessary to fine-tune and locate optimal defect distributions, which could be the key to increasing the success rate of the realization of QAHE. Such tuning can be done by varying growth conditions, including synthesis methods, growth temperature, time, chemical environment and post-annealing effect. We have seen that Sb or Pb doping indeed modified antisite formation. In addition, Na doping on the Mn site was predicted to be an effective doping that only reduces Bi_Mn_ [82], which awaits experimental verification. It also remains an open question if single-crystal growth under high pressure can make the SL and QL a better lattice match to reduce the number of antisites.

The general material design principles of natural heterostructural MnBi_2*n*_Te_3*n*+1_ are not limited to this material family. Knowledge of the growth of metastable phases, understanding the effect of defects in magnetism and topology, etc. have flourished with the tremendous efforts in studies of natural heterostructural materials. The research in this aspect can serve as a guide to growing more intrinsic magnetic topological materials. Examples include Mn_*n*_Bi_2_Te_3 + *n*_, where more than one MnTe layer is embedded within each slab, was proposed [104] and observed in thin-film growth for *n* = 3 [53]. Recently, Mn_2_Bi_2_Te_5_ has also been identified [62,105] in the bulk sample, although it only comes in as a small amount of impurity phase of MnBi_2_Te_4_. This entails, by now unsurprisingly, more delicate metastable magnetic topological insulator phases awaiting us to explore. Ongoing research is needed to continue optimizing MnBi_2*n*_Te_3*n*+1_ and search for more MTI candidates to provide a fertile ground for the interplay between topology and magnetism and the realization of QAHE at higher temperatures with good reproducibility.

## References

[1] Zhang D , Shi M, Zhu T et al. Topological axion states in the magnetic insulator MnBi_2_Te_4_ with the quantized magnetoelectric effect. Phys Rev Lett 2019; 122: 206401. 10.1103/PhysRevLett.122.206401 31172761

[2] Li J , Li Y, Du S et al. Intrinsic magnetic topological insulators in van der Waals layered MnBi_2_Te_4_-family materials. Sci Adv 2019; 5: eaaw5685. 10.1126/sciadv.aaw5685 31214654 PMC6570506

[3] Otrokov MM , Klimovskikh II, Bentmann H et al. Prediction and observation of an antiferromagnetic topological insulator. Nature 2019; 576: 416–22. 10.1038/s41586-019-1840-9 31853084

[4] Deng Y , Yu Y, Shi MZ et al. Quantum anomalous Hall effect in intrinsic magnetic topological insulator MnBi_2_Te_4_. Science 2020; 367: 895–900. 10.1126/science.aax8156 31974160

[5] Liu C , Wang Y, Li H et al. Robust axion insulator and chern insulator phases in a two-dimensional antiferromagnetic topological insulator. Nat Mater 2020; 19: 522–7. 10.1038/s41563-019-0573-3 31907415

[6] Ge J , Liu Y, Li J et al. High-Chern-number and high-temperature quantum Hall effect without Landau levels. Natl Sci Rev 2020; 7: 1280–7. 10.1093/nsr/nwaa089 34692156 PMC8289033

[7] He K , Wang Y, Xue QK. Topological materials: quantum anomalous Hall system. Annu Rev Condens Matter Phys 2018; 9: 329–44. 10.1146/annurev-conmatphys-033117-054144

[8] Liu CX , Zhang SC, Qi XL. The quantum anomalous Hall effect: theory and experiment. Annu Rev Condens Matter Phys 2016; 7: 301–21. 10.1146/annurev-conmatphys-031115-011417

[9] Wang J , Lian B, Qi XL et al. Quantized topological magnetoelectric effect of the zero-plateau quantum anomalous Hall state. Phys Rev B 2015; 92: 081107. 10.1103/PhysRevB.92.081107

[10] Tokura Y , Yasuda K, Tsukazaki A. Magnetic topological insulators. Nat Rev Phys 2019; 1: 126–43. 10.1038/s42254-018-0011-5

[11] Chang CZ , Zhang J, Feng X et al. Experimental observation of the quantum anomalous Hall effect in a magnetic topological insulator. Science 2013; 340: 167–70. 10.1126/science.1234414 23493424

[12] Lee DS , Kim TH, Park CH et al. Crystal structure, properties and nanostructuring of a new layered chalcogenide semiconductor, Bi_2_MnTe_4_. CrystEngComm 2013; 15: 5532–8. 10.1039/c3ce40643a

[13] Aliev ZS , Amiraslanov IR, Nasonova DI et al. Novel ternary layered manganese bismuth tellurides of the MnTe-Bi_2_Te_3_ system: synthesis and crystal structure. J Alloys Compd 2019; 789: 443–50. 10.1016/j.jallcom.2019.03.030

[14] Gong Y , Guo J, Li J et al. Experimental realization of an intrinsic magnetic topological insulator. Chin Phys Lett 2019; 36: 076801. 10.1088/0256-307X/36/7/076801

[15] Yan JQ , Zhang Q, Heitmann T et al. Crystal growth and magnetic structure of MnBi_2_Te_4_. Phys Rev Mater 2019; 3: 064202. 10.1103/PhysRevMaterials.3.064202

[16] Zeugner A , Nietschke F, Wolter AU et al. Chemical aspects of the candidate antiferromagnetic topological insulator MnBi_2_Te_4_. Chem Mater 2019; 31: 2795–806. 10.1021/acs.chemmater.8b05017

[17] Lee SH , Zhu Y, Wang Y et al. Spin scattering and noncollinear spin structure-induced intrinsic anomalous Hall effect in antiferromagnetic topological insulator MnBi_2_Te_4_. Phys Rev Res 2019; 1: 012011. 10.1103/PhysRevResearch.1.012011 PMC13244286 42266839

[18] Otrokov MM , Rusinov IP, Blanco-Rey M et al. Unique thickness-dependent properties of the van der Waals interlayer antiferromagnet MnBi_2_Te_4_ films. Phys Rev Lett 2019; 122: 107202. 10.1103/PhysRevLett.122.107202 30932645

[19] Chen B , Fei F, Zhang D et al. Intrinsic magnetic topological insulator phases in the Sb doped MnBi_2_Te_4_ bulks and thin flakes. Nat Commun 2019; 10: 1–8. 10.1038/s41467-019-12485-y 31578337 PMC6775157

[20] Hao YJ , Liu P, Feng Y et al. Gapless surface dirac cone in antiferromagnetic topological insulator MnBi_2_Te_4_. Phys Rev X 2019; 9: 041038. 10.1103/PhysRevX.9.041038

[21] Chen Y , Xu L, Li J et al. Topological electronic structure and its temperature evolution in antiferromagnetic topological insulator MnBi_2_Te_4_. Phys Rev X 2019; 9: 041040. 10.1103/PhysRevX.9.041040

[22] Li H , Gao SY, Duan SF et al. Dirac surface states in intrinsic magnetic topological insulators EuSn_2_As_2_ and MnBi_2*n*_Te_3*n*+1_. Phys Rev X 2019; 9: 041039. 10.1103/PhysRevX.9.041039

[23] Li B , Yan JQ, Pajerowski DM et al. Competing magnetic interactions in the antiferromagnetic topological insulator MnBi_2_Te_4_. Phys Rev Lett 2020; 124: 167204. 10.1103/PhysRevLett.124.167204 32383954

[24] Li H , Liu S, Liu C et al. Antiferromagnetic topological insulator MnBi_2_Te_4_: synthesis and magnetic properties. Phys Chem Chem Phys 2020; 22: 556–63. 10.1039/C9CP05634C 31840700

[25] Ding L , Hu C, Ye F et al. Crystal and magnetic structures of magnetic topological insulators MnBi_2_Te_4_ and MnBi_4_Te_7_. Phys Rev B 2020; 101: 020412. 10.1103/PhysRevB.101.020412

[26] Gao A , Liu YF, Hu C et al. Layer Hall effect in a 2D topological axion antiferromagnet. Nature 2021; 595: 521–5. 10.1038/s41586-021-03679-w 34290425

[27] Ovchinnikov D , Huang X, Lin Z et al. Intertwined topological and magnetic orders in atomically thin Chern insulator MnBi_2_Te_4_. Nano Lett 2021; 21: 2544–50. 10.1021/acs.nanolett.0c05117 33710884

[28] Hu C , Gordon KN, Liu P et al. A van der Waals antiferromagnetic topological insulator with weak interlayer magnetic coupling. Nat Commun 2020; 11: 97. 10.1038/s41467-019-13814-x 31911588 PMC6946652

[29] Tian S , Gao S, Nie S et al. Magnetic topological insulator MnBi_6_Te_10_ with a zero-field ferromagnetic state and gapped dirac surface states. Phys Rev B 2020; 102: 035144. 10.1103/PhysRevB.102.035144

[30] Hu C , Ding L, Gordon KN et al. Realization of an intrinsic ferromagnetic topological state in MnBi_8_Te_13_. Sci Adv 2020; 6: eaba4275. 10.1126/sciadv.aba4275 32743072 PMC7375807

[31] Deng H , Chen Z, Wołoś A et al. High-temperature quantum anomalous Hall regime in a MnBi_2_Te_4_/Bi_2_Te_3_ superlattice. Nat Phys 2021; 17: 36–42. 10.1038/s41567-020-0998-2

[32] Shi M , Lei B, Zhu C et al. Magnetic and transport properties in the magnetic topological insulators MnBi_2_Te_4_(Bi_2_Te_3_)_*n*_ (*n* = 1, 2). Phys Rev B 2019; 100: 155144. 10.1103/PhysRevB.100.155144

[33] Sun H , Xia B, Chen Z et al. Rational design principles of the quantum anomalous Hall effect in superlatticelike magnetic topological insulators. Phys Rev Lett 2019; 123: 096401. 10.1103/PhysRevLett.123.096401 31524481

[34] Ding L , Hu C, Feng E et al. Neutron diffraction study of magnetism in van der Waals layered MnBi_2*n*_Te_3*n*+1_. J Phys D 2021; 54: 174003. 10.1088/1361-6463/abe0dd

[35] Wu J , Liu F, Sasase M et al. Natural van der Waals heterostructural single crystals with both magnetic and topological properties. Sci Adv 2019; 5: eaax9989. 10.1126/sciadv.aax9989 31763457 PMC6858254

[36] Yan JQ , Liu Y, Parker D et al. A-type antiferromagnetic order in MnBi_4_Te_7_ and MnBi_6_Te_10_ single crystals. Phys Rev Mater 2020; 4: 054202. 10.1103/PhysRevMaterials.4.054202

[37] Gordon KN, Sun H and Hu C et al. Strongly gapped topological surface states on protected surfaces of antiferromagnetic MnBi_4_Te_7_ and MnBi_6_Te_10_, arXiv, 2019. https://arxiv.org/abs/1910.13943

[38] Souchay D , Nentwig M, Günther D et al. Layered manganese bismuth tellurides with GeBi_4_Te_7_-and GeBi_6_Te_10_-type structures: towards multifunctional materials. J Mater Chem C 2019; 7: 9939–53. 10.1039/C9TC00979E

[39] Hu Y , Xu L, Shi M et al. Universal gapless Dirac cone and tunable topological states in (MnBi_2_Te_4_)_*m*_(Bi_2_Te_3_)_*n*_ heterostructures. Phys Rev B 2020; 101: 161113. 10.1103/PhysRevB.101.161113

[40] Xu L , Mao Y, Wang H et al. Persistent surface states with diminishing gap in MnBi_2_Te_4_/Bi_2_Te_3_ superlattice antiferromagnetic topological insulator. Sci Bull 2020; 65: 2086–93. 10.1016/j.scib.2020.07.032 36732961

[41] Jo NH , Wang LL, Slager RJ et al. Intrinsic axion insulating behavior in antiferromagnetic MnBi_6_Te_10_. Phys Rev B 2020; 102: 045130. 10.1103/PhysRevB.102.045130

[42] Rienks E , Wimmer S, Sánchez Barriga J et al. Large magnetic gap at the Dirac point in Bi_2_Te_3_/MnBi_2_Te_4_ heterostructures. Nature 2019; 576: 423–8. 10.1038/s41586-019-1826-7 31853081

[43] Tan A , Labracherie V, Kunchur N et al. Metamagnetism of weakly coupled antiferromagnetic topological insulators. Phys Rev Lett 2020; 124:197201. 10.1103/PhysRevLett.124.197201 32469595

[44] Klimovskikh II , Otrokov MM, Estyunin D et al. Tunable 3D/2D magnetism in the (MnBi_2_Te_4_)(Bi_2_Te_3_)_*m*_ topological insulators family. npj Quantum Mater 2020; 5: 1–9.

[45] Vidal R , Bentmann H, Facio J et al. Orbital complexity in intrinsic magnetic topological insulators MnBi_4_Te_7_ and MnBi_6_Te_10_. Phys Rev Lett 2021; 126: 176403. 10.1103/PhysRevLett.126.176403 33988442

[46] Lu R , Sun H, Kumar S et al. Half-magnetic topological insulator with magnetization-induced Dirac gap at a selected surface. Phys Rev X 2021; 11: 011039. 10.1103/PhysRevX.11.011039

[47] Zhong H , Bao C, Wang H et al. Light-tunable surface state and hybridization gap in magnetic topological insulator MnBi_8_Te_13_. Nano Lett 2021; 21: 6080–6. 10.1021/acs.nanolett.1c01448 34242038

[48] Lee SH , Graf D, Min L et al. Evidence for a magnetic-field-induced ideal type-II Weyl state in antiferromagnetic topological insulator Mn(Bi_1 −*x*_Sb_*x*_)_2_Te_4_. Phys Rev X 2021; 11: 031032. 10.1103/PhysRevX.11.031032

[49] Lee SH , Graf D, Robinson R et al. Evidence of magnetic fluctuation induced Weyl semimetal state in the antiferromagnetic topological insulator Mn(Bi_1 −*x*_Sb_*x*_)_2_Te_4_. Phys Rev B 2023; 107: 205105. 10.1103/PhysRevB.107.205105

[50] Jiang Q, Palmstrom JC and Singleton J et al. Fermi surface evolution and anomalous Hall effect in an ideal type-II Weyl semimetal, arXiv, 2023. https://arxiv.org/abs/2306.08339

[51] Hu C , Lien SW, Feng E et al. Tuning magnetism and band topology through antisite defects in Sb-doped MnBi_4_Te_7_. Phys Rev B 2021; 104: 054422. 10.1103/PhysRevB.104.054422

[52] Hirahara T , Eremeev SV, Shirasawa T et al. Large-gap magnetic topological heterostructure formed by subsurface incorporation of a ferromagnetic layer. Nano Lett 2017; 17: 3493–500. 10.1021/acs.nanolett.7b00560 28545300

[53] Hirahara T , Otrokov MM, Sasaki T et al. Fabrication of a novel magnetic topological heterostructure and temperature evolution of its massive Dirac cone. Nat Commun 2020; 11: 4821. 10.1038/s41467-020-18645-9 32973165 PMC7515900

[54] Zhu T , Bishop AJ, Zhou T et al. Synthesis, magnetic properties, and electronic structure of magnetic topological insulator MnBi_2_Se_4_. Nano Lett 2021; 21: 5083–90. 10.1021/acs.nanolett.1c00141 34097421

[55] Zhao YF , Zhou LJ, Wang F et al. Even–odd layer-dependent anomalous Hall effect in topological magnet MnBi_2_Te_4_ thin films. Nano Lett. 2021; 21: 7691–8. 10.1021/acs.nanolett.1c02493 34468149

[56] Liu M , Lei C, Kim H et al. Visualizing the interplay of Dirac mass gap and magnetism at nanoscale in intrinsic magnetic topological insulators. Proc Natl Acad Sci USA 2022; 119: e2207681119. 10.1073/pnas.2207681119 36215491 PMC9586289

[57] Yu S , Zhao K, Yang X et al. The synthesis of MnBi_2_Te_4_ antiferromagnetic topological insulator single crystals through a one-step growth method. J Supercond Nov Magn 2022; 35: 1221–8. 10.1007/s10948-022-06175-y

[58] Hu C , Gao A, Berggren BS et al. Growth, characterization, and Chern insulator state in MnBi_2_Te_4_ via the chemical vapor transport method. Phys Rev Mater 2021; 5: 124206. 10.1103/PhysRevMaterials.5.124206

[59] Cui J , Shi M, Wang H et al. Transport properties of thin flakes of the antiferromagnetic topological insulator MnBi_2_Te_4_. Phys Rev B 2019; 99: 155125. 10.1103/PhysRevB.99.155125

[60] Amiraslanov I , Aliev Z, Askerova P et al. Crystal structure and Raman-active lattice vibrations of magnetic topological insulators MnBi_2_Te_4_·*n*(Bi_2_Te_3_) (*n* = 0, 1, …, 6). Phys Rev B 2022; 106: 184108. 10.1103/PhysRevB.106.184108

[61] Canfield PC , Fisk Z. Growth of single crystals from metallic fluxes. Philos Mag B 1992; 65: 1117–23. 10.1080/13642819208215073

[62] Yan JQ , Huang Z, Wu W et al. Vapor transport growth of MnBi_2_Te_4_ and related compounds. J Alloys Compd 2022; 906: 164327. 10.1016/j.jallcom.2022.164327

[63] Orujlu E , Aliev Z, Amiraslanov I et al. Phase equilibria of the MnTe-Sb_2_Te_3_ system and synthesis of novel ternary layered compound–MnSb_4_Te_7_. Phys Chem Solid State 2021; 22: 39–44. 10.15330/pcss.22.1.39-44

[64] Zhu J , Naveed M, Chen B et al. Magnetic and electrical transport study of the antiferromagnetic topological insulator Sn-doped MnBi_2_Te_4_. Phys Rev B 2021; 103: 144407. 10.1103/PhysRevB.103.144407

[65] Qian T , Yao YT, Hu C et al. Magnetic dilution effect and topological phase transitions in (Mn_1 −*x*_Pb_*x*_)Bi_2_Te_4_. Phys Rev B 2022; 106: 045121. 10.1103/PhysRevB.106.045121

[66] Yan JQ , Okamoto S, McGuire MA et al. Evolution of structural, magnetic, and transport properties in MnBi_2 −*x*_Sb_*x*_Te_4_. Phys Rev B 2019; 100: 104409. 10.1103/PhysRevB.100.104409

[67] Qian T , Emmanouilidour E, Hu C et al. Unconventional pressure-driven metamagnetic transitions in topological van der Waals magnets. Nano Lett 2022; 22: 5523–9. 10.1021/acs.nanolett.2c01680 35731986

[68] Wu J , Liu F, Liu C et al. Toward 2D magnets in the (MnBi_2_Te_4_)(Bi_2_Te_3_)_*n*_ bulk crystal. Adv Mater. 2020; 32: 2001815. 10.1002/adma.202001815 32329547

[69] Hu C , Tanatar MA, Prozorov R et al. Unusual dynamic susceptibility arising from soft ferromagnetic domains in MnBi_8_Te_13_ and Sb-doped MnBi_2*n*_Te_3*n*+1_ (*n* = 2, 3). J Phys D 2021; 55: 054003. 10.1088/1361-6463/ac3032

[70] Guan YD , Yan CH, Lee SH et al. Ferromagnetic MnBi_4_Te_7_ obtained with low-concentration Sb doping: a promising platform for exploring topological quantum states. Phys Rev Mater 2022; 6: 054203. 10.1103/PhysRevMaterials.6.054203

[71] Li H , Li Y, Lian Y et al. Glassy magnetic ground state in layered compound MnSb_2_Te_4_. Sci China Mater 2022; 65: 477–85. 10.1007/s40843-021-1738-9

[72] Yan C , Zhu Y, Miao L et al. Delicate ferromagnetism in MnBi_6_Te_10_. Nano Lett 2022; 22: 9815–22. 10.1021/acs.nanolett.2c02500 36315185 PMC9801432

[73] Scholten M , Facio JI, Ray R et al. Finite temperature fluctuation-induced order and responses in magnetic topological insulators. Phys Rev Res 2021; 3: L032014. 10.1103/PhysRevResearch.3.L032014

[74] Alfonsov A , Mehlawat K, Zeugner A et al. Magnetic-field tuning of the spin dynamics in the magnetic topological insulators (MnBi_2_Te_4_)(Bi_2_Te_3_)_*n*_. Phys Rev B 2021; 104: 195139. 10.1103/PhysRevB.104.195139

[75] McLaughlin NJ , Hu C, Huang M et al. Quantum imaging of magnetic phase transitions and spin fluctuations in intrinsic magnetic topological nanoflakes. Nano Lett 2022; 22: 5810–7. 10.1021/acs.nanolett.2c01390 35816128

[76] Yan JQ . Perspective–the elusive quantum anomalous Hall effect in MnBi_2_Te_4_: materials. ECS J Solid State Sci Technol 2022; 11: 063007. 10.1149/2162-8777/ac70fc

[77] Hou F , Yao Q, Zhou CS et al. Te-vacancy-induced surface collapse and reconstruction in antiferromagnetic topological insulator MnBi_2_Te_4_. ACS nano 2020; 14: 11262–72. 10.1021/acsnano.0c03149 32813492

[78] Huang Z , Du MH, Yan J et al. Native defects in antiferromagnetic topological insulator MnBi_2_Te_4_. Phys Rev Mater 2020; 4: 121202. 10.1103/PhysRevMaterials.4.121202

[79] Yuan Y , Wang X, Li H et al. Electronic states and magnetic response of MnBi_2_Te_4_ by scanning tunneling microscopy and spectroscopy. Nano Lett 2020; 20: 3271–7. 10.1021/acs.nanolett.0c00031 32298117

[80] Wu X , Li J, Ma XM et al. Distinct topological surface states on the two terminations of MnBi_4_Te_7_. Phys Rev X 2020; 10: 031013. 10.1103/PhysRevX.10.031013

[81] Liang Z , Luo A, Shi M et al. Mapping Dirac fermions in the intrinsic antiferromagnetic topological insulators (MnBi_2_Te_4_)(Bi_2_Te_3_)_*n*_ (*n* = 0, 1). Phys Rev B 2020; 102: 161115. 10.1103/PhysRevB.102.161115

[82] Liu Y , Wang LL, Zheng Q et al. Site mixing for engineering magnetic topological insulators. Phys Rev X 2021; 11: 021033. 10.1103/PhysRevX.11.021033

[83] Lai Y , Ke L, Yan J et al. Defect-driven ferrimagnetism and hidden magnetization in MnBi_2_Te_4_. Phys Rev B 2021; 103: 184429. 10.1103/PhysRevB.103.184429

[84] Riberolles SXM , Zhang Q, Gordon E et al. Evolution of magnetic interactions in Sb-substituted MnBi_2_Te_4_. Phys Rev B 2021; 104: 064401. 10.1103/PhysRevB.104.064401

[85] Du MH , Yan J, Cooper VR et al. Tuning Fermi levels in intrinsic antiferromagnetic topological insulators MnBi_2_Te_4_ and MnBi_4_Te_7_ by defect engineering and chemical doping. Adv Funct Mater 2021; 31: 2006516.

[86] Garnica M , Otrokov MM, Aguilar PC et al. Native point defects and their implications for the Dirac point gap at MnBi_2_Te_4_ (0001). npj Quantum Mater 2022; 7: 1–9. 10.1038/s41535-021-00414-6

[87] Chen K , Wang B, Yan JQ et al. Suppression of the antiferromagnetic metallic state in the pressurized MnBi_2_Te_4_ single crystal. Phys Rev Mater 2019; 3: 094201. 10.1103/PhysRevMaterials.3.094201

[88] Chen B , Wang D, Jiang Z et al. Coexistence of ferromagnetism and topology by charge carrier engineering in the intrinsic magnetic topological insulator MnBi_4_Te_7_. Phys Rev B 2021; 104: 075134. 10.1103/PhysRevB.104.075134

[89] Xie H , Fei F, Fang F et al. Charge carrier mediation and ferromagnetism induced in MnBi_6_Te_10_ magnetic topological insulators by antimony doping. J Phys D 2021; 55: 104002.

[90] Murakami T , Nambu Y, Koretsune T et al. Realization of interlayer ferromagnetic interaction in MnSb_2_Te_4_ toward the magnetic Weyl semimetal state. Phys Rev B 2019; 100: 195103. 10.1103/PhysRevB.100.195103 PMC7919059 33655090

[91] Chen Y , Chuang YW, Lee SH et al. Ferromagnetism in van der Waals compound MnSb_1.8_Bi_0.2_Te_4_. Phys Rev Mater 2020; 4: 064411. 10.1103/PhysRevMaterials.4.064411

[92] Shi G , Zhang M, Yan D et al. Anomalous Hall effect in layered ferrimagnet MnSb_2_Te_4_. Chin Phys Lett 2020; 37: 047301. 10.1088/0256-307X/37/4/047301

[93] Ge W , Sass PM, Yan J et al. Direct evidence of ferromagnetism in MnSb_2_Te_4_. Phys Rev B 2021; 103: 134403. 10.1103/PhysRevB.103.134403

[94] Wimmer S , Sánchez-Barriga J, Küppers P et al. Mn-rich MnSb_2_Te_4_: a topological insulator with magnetic gap closing at high Curie temperatures of 45–50 k. Adv Mater 2021; 33: 2102935. 10.1002/adma.202102935 PMC11468489 34469013

[95] Chong SK , Lei C, Lee SH et al. Anomalous Landau quantization in intrinsic magnetic topological insulators. Nat Commun 2023; 14: 4805. 10.1038/s41467-023-40383-x 37558682 PMC10412595

[96] Alexandradinata A , Wang C, Duan W et al. Revealing the topology of Fermi-surface wave functions from magnetic quantum oscillations. Phys Rev X 2018; 8: 011027. 10.1103/PhysRevX.8.011027

[97] Jiang Q , Wang C, Malinowski P et al. Quantum oscillations in the field-induced ferromagnetic state of MnBi_2 −*x*_Sb_*x*_Te_4_. Phys Rev B 2021; 103: 205111. 10.1103/PhysRevB.103.205111

[98] Zyuzin AA , Tiwari RP. Intrinsic anomalous Hall effect in type-II Weyl semimetals. JETP Lett 2016; 103: 717–22. 10.1134/S002136401611014X

[99] Huan S , Zhang S, Jiang Z et al. Multiple magnetic topological phases in bulk van der Waals crystal MnSb_4_Te_7_. Phys Rev Lett 2021; 126: 246601. 10.1103/PhysRevLett.126.246601 34213928

[100] Pei C , Xia Y, Wu J et al. Pressure-induced topological and structural phase transitions in an antiferromagnetic topological insulator. Chin Phys Lett 2020; 37: 066401. 10.1088/0256-307X/37/6/066401

[101] Shao J , Liu Y, Zeng M et al. Pressure-tuned intralayer exchange in superlattice-like MnBi_2_Te_4_/(Bi_2_Te_3_)_*n*_ topological insulators. Nano Lett 2021; 21: 5874–80. 10.1021/acs.nanolett.1c01874 34197120

[102] Yin Y , Ma X, Yan D et al. Pressure-driven electronic and structural phase transition in intrinsic magnetic topological insulator MnSb_2_Te_4_. Phys Rev B 2021; 104: 174114. 10.1103/PhysRevB.104.174114

[103] Pei C , Xi M, Wang Q et al. Pressure-induced superconductivity in magnetic topological insulator candidate MnSb_4_Te_7_. Phys Rev Mater 2022; 6: L101801. 10.1103/PhysRevMaterials.6.L101801

[104] Eremeev SV , Otrokov MM, Chulkov EV. New universal type of interface in the magnetic insulator/topological insulator heterostructures. Nano Lett 2018; 18: 6521–9. 10.1021/acs.nanolett.8b03057 30260648

[105] Cao L , Han S, Lv YY et al. Growth and characterization of the dynamical axion insulator candidate Mn_2_Bi_2_Te_5_ with intrinsic antiferromagnetism. Phys Rev B 2021; 104: 054421. 10.1103/PhysRevB.104.054421

